# Characteristics of *dr1790* disruptant and its functional analysis in *Deinococcus radiodurans*

**DOI:** 10.1590/S1517-838246220131018

**Published:** 2015-06-01

**Authors:** Jianhui Cheng, Hu Wang, Xin Xu, Liangyan Wang, Bing Tian, Yuejin Hua

**Affiliations:** 1 Institute of Horticulture, Zhejiang Academy of Agricultural Sciences, Hangzhou, China, Institute of Horticulture, Zhejiang Academy of Agricultural Sciences, Hangzhou, China.; 2 Zhejiang University, Institute of Nuclear-Agricultural Sciences, Zhejiang University, Hangzhou, China, Institute of Nuclear-Agricultural Sciences, Zhejiang University, Hangzhou, China.; 3 Institute of Ageing Research, Hangzhou Normal University, Hangzhou, China, Institute of Ageing Research, Hangzhou Normal University, Hangzhou, China.

**Keywords:** *Deinococcus radiodurans*, *dr1790* disruptant, characteristics, functional analysis

## Abstract

*Deinococcus radiodurans* (DR) is an extremophile that is well known for its resistance to radiation, oxidants and desiccation. The gene *dr1790* of *D*. *radiodurans* was predicted to encode a yellow-related protein. The primary objective of the present study was to characterize the biological function of the DR1790 protein, which is a member of the ancient yellow/major royal jelly (MRJ) protein family, in prokaryotes. Fluorescence labeling demonstrated that the yellow-related protein encoded by *dr1790* is a membrane protein. The deletion of the *dr1790* gene decreased the cell growth rate and sensitivity to hydrogen peroxide and radiation and increased the membrane permeability of *D*. *radiodurans*. Transcript profiling by microarray and RT-PCR analyses of the *dr1790* deletion mutant suggested that some genes that are involved in protein secretion and transport were strongly suppressed, while other genes that are involved in protein quality control, such as chaperones and proteases, were induced. In addition, the expression of genes with predicted functions that are involved in antioxidant systems, electron transport, and energy metabolism was significantly altered through the disruption of *dr1790*. Moreover, the results of proteomic analyses using 2-DE and MS also demonstrated that DR1790 contributed to *D*. *radiodurans* survival. Taken together, these results indicate that the DR1790 protein from the ancient yellow protein family plays a pleiotropic role in the survival of prokaryotic cells and contributes to the extraordinary resistance of *D. radiodurans* against oxidative and radiation stresses.

## Introduction

D. *radiodurans* exhibits resistance to the lethal and mutagenic effects of DNA damaging agents, including γ-ray and UV radiation, hydrogen peroxide and desiccation (Battista, 1997; Makarova *et al.*, 2001; Shu and Tian, 2010; Ghosal *et al.*, 2005). These bacteria can survive ~12 kGy γ-ray irradiation, which generates approximately 200 double-strand and 3000 single-strand breaks per genome (Battista, 2000). The robustness of this bacterium reflects strong oxidative stress resistance mechanisms that protect proteins from oxidative damage (Wang *et al.*, 1995; Markillie *et al.*, 1999; Daly *et al.*, 2007) and a DNA repair process that efficiently and precisely reassembles DNA fragments (Minton 1994; Slade *et al.*, 2009). Antioxidant protection and repair mechanisms for DNA and other proteins enable these molecules to retain their catalytic activity and to provide a swift response under oxidative stress conditions (Slade and Radman, 2011). Genetic engineering techniques may be applied to *D. radiodurans*, which has extreme resistance, as well as the ability to self-repair DNA damage, to bioremediate radioactive waste sites, to breed plants for resistance and to treat human cancer. Therefore, *D. radiodurans*, which is of interest to many researchers, represents a microbial resource with great development prospects. *D. radiodurans* strains that express the cloned Hg(II) resistance gene (*merA*) from the *E. coli* strain BL308 exhibit growth in the presence of both 60 Gy/h of ^137^Cs radiation (a dose rate that exceeds those in most radioactive waste sites) and 30–50 μM Hg(II) and that effectively reduce Hg(II) to the less toxic volatile elemental Hg(0) (Brim *et al.*, 2000). The cloning of toluene dioxygenase *tod* genes from *Pseudomonas putida* F1 into the chromosome of *D. radiodurans* conferred the ability to oxidize toluene, chlorobenzene, 3,4-dichloro-1-butene, and indole in a highly irradiating environment (Lange *et al.*, 1998). The expression of *IrrE*, which is a global regulator for extreme radiation resistance in *D. radiodurans*, significantly enhanced salt tolerance in *Brassica napus* plants. Transgenic *B. napus* plants that express the *IrrE* can tolerate 350 mM NaCl, which is a concentration that inhibits the growth of almost all crop plants (Pan *et al.*, 2009). The human bone marrow cell line KG1a, which was transformed with *dr1709* from *D. radiodurans*, exhibited a much higher survival fraction than the original KG1a cells when treated with γ-ray radiation (Shu and Tian, 2012). However, the underlying mechanisms of *D. radiodurans* resistance to stresses remain unclear. Therefore, the identification and functional analysis of new genes that are associated with anti-radiation, DNA repair and antioxidants will improve our understanding of the extreme radiation resistance mechanisms of this strain and provide strategies for research regarding the radiation damage defense and oxidative stress resistance systems of organisms.

*dr1790*, which is a gene that encodes a putative yellow-related protein homolog, was identified in the *D. radiodurans* genome (Makarova *et al.*, 2001). Interestingly, this yellow-related protein is typically detected in insects and plays important roles in pigmentation and insect behaviors. The deletion of the yellow protein gene locus in *Drosophila* not only affects larval pigmentation but also appears to affect insect behavior (Maleszka and Kucharski, 2000; Drapeau et *al*., 2006). Yellow protein can be secreted from cells because this protein contains a secretion signal peptide (Drapeau, 2003). Furthermore, other members of the Yellow/Major Royal Jelly (MRJ) protein family are expressed in not only insects but also some bacterial and fungi species, suggesting that yellow proteins are evolutionarily ancient (Drapeau *et al.*, 2006). Although a few studies have demonstrated an association between melanization and behavior in *Drosophila*, and a unique clade of genes from *Apis mellifera* may be involved in caste specification, the function of most yellow protein family members remains largely unknown (Ferguson *et al.*, 2011). Currently, no studies concerning the function of yellow-related proteins in prokaryotes exist. DR1790 expression was induced in a *D. radiodurans* mutant strain that was deficient in OxyR, which is a peroxide sensor and transcription regulator that senses the presence of reactive oxygen species and that induces the antioxidant system of *D*. *radiodurans* (Chen *et al.*, 2008). These findings prompted us to investigate the functions of this yellow-related protein homolog in this extremophilic bacterium*.*

## Materials and Methods

### Bacterial strains and materials

All *D. radiodurans* cultures were grown at 30 °C in tryptone-yeast extract-glucose (TGY) media (0.5% bacto-tryptone, 0.3% bacto-yeast extract, and 0.1% glucose) with aeration or on TGY plates solidified with 1.5% agar. Overnight cultures were incubated in fresh TGY medium, and exponential-phase cells (OD_600nm_ = 0.8) were used for all experiments. The *E. coli* strain JM109 was grown in Luria-Bertani (LB) broth (1.0% bacto-tryptone, 0.5% bacto-yeast extract, and 1.0% NaCl) or on LB plates solidified with 1.5% agar at 37 °C.

### Construction of mutant strains

The *D. radiodurans* strain R1Δ*dr1790* was constructed using a deletion replacement method as described previously (Xu *et al.*, 2008). The primers that were used in this study are listed in Table 1. The primers p1 and p2 were used to amplify a *Bam*HI fragment upstream of the targeted genes, and the primers p3 and p4 were used to amplify a *Hind*III fragment downstream of the targeted genes. The kanamycin resistance cassette containing the *GroEL* promoter was obtained from the pRADK shuttle plasmid (Gao *et al.*, 2005). The three DNA fragments were digested and ligated; then, the ligation products were used as templates for PCR (30 cycles at 94 °C for 1 min, 55 °C for 45 s, and 72 °C for 1 min) with p1 and p4. The resulting PCR fragments were transformed into *D. radiodurans* cells using the CaCl_2_ technique, and the mutant strains were selected on TGY agar plates supplemented with 20 μg/mL kanamycin.

**Table 1:** Primers used in this study.

Primer	Sequence
Construction of the R1Δ*dr1790* mutant	
p1	5′ GGTGTGTTTGACTGAGGCCGAGGAC 3′
p2	5′ GTTGGATCCCAGGGGTATAAGACGC 3′
p3	5′ TTTAAGCTTGCTGCACGTTGACCCT 3′
p4	5′ TGTTGTGTTGCCTACCTGGCGATTG 3′
Kanamycin F	5′ CACACAGGAAACAGCTATGACCATGATTA 3′
Kanamycin R	5′ ACAGACGGATCCTAGAAAAACTCATCGAGCATC 3′
Complementation of the R1Δ*dr1790* mutant	
DR1790_com_ F	5′ TTTCATATGATGAAAATCAAGCTGACCGC 3′
DR1790_com_ R	5′ TTTGGATCCTTATTTCAGCAGCACCGGC 3′
Real-time quantitative PCR	
DR0089	F: 5′ TACCGCTCTTACCCCGACTC 3′
	R: 5′ CGTGTAGATGGCGAACACCA 3′
DR0126	F: 5′ TGACGACTACGGTGGATGTGC 3′
	R: 5′ CTCGTCGCTGAGGTCTTTGG 3′
DR0128	F: 5′ GCAACCGCACCACCATCG 3′
	R: 5′ TTCGTCTTCGTCACCAGCAAC 3′
DR0129	F: 5′ CGCAAGGGCAACGAAACTG 3′
	R: 5′ GGTGATGAAGGGCAGGGAGAT 3′
DR0194	F: 5′ CTCACCGACCACTACGACCCG 3′
	R: 5′ CGCCCCGCCGAACAGAAT 3′
DR0350	F: 5′ CAGATAGCCACGCTCAACGC 3′
	R: 5′ CGACCCGGAAGCCCTTTT 3′
DR0606	F: 5′ CGAAGAAGCCGAGCAGAAGA 3′
	R: 5′ GGTGCCGTTGTCCAGGGTC 3′
DR0607	F: 5′ AGCACCGACTCCGACTACGC 3′
	R: 5′ GCCTGCCACGATGCCTTCT 3′
DR0888	F: 5′ AGGTGACGGGTGAGGTGGC 3′
	R: 5′ GCTGGGGCTGGTTTGTGC 3′
DR1046	F: 5′ CGGCGACAGTTTCGTGGC 3′
	R: 5′ GCTGTTCACTGGTTTTGTTGGTC 3′
DR1114	F: 5′ CCCCGAACTTCACTCCCA 3′
	R: 5′ CGGTCAGGGTCTGGTTTTCA 3′
DR1148	F: 5′ CATATGGTTTTTCATGGACGGCTCC3′
	R: 5′ GGATCCTCAAGAGTCGGCCCCGCTA3′
DR1172	F: 5′ GTCTGTTGCTGCTCGGTGCC 3′
	R: 5′ TGGTCTTTTCCCAGCCCTTG 3′
DR1909	F: 5′ GCCTACACGCACGTTTCCG 3′
	R: 5′ CCTCACGCACCACGCAGA 3′
DR1974	F: 5′ GCCACCTGGACCCCTGAG 3′
	R: 5′ GCATTCCGGCTTCTTCGAT 3′

### Complementation of R1Δ*dr1790*

The complementation plasmid was constructed as described previously (Gao *et al.*, 2005; Wu *et al.*, 2009). Briefly, chromosomal DNA was isolated from wild type strains. The 1167-bp region containing the *dr1790* gene was PCR-amplified (35 cycles at 94 °C for 1 min, 58 °C for 50 s and 72 °C for 1 min) using the primers DR1790_com_F and DR1790_com_R (Table 1) and ligated into the pMD18 T-Easy vector (Takara, JP); the resulting construct was designated as pMD-*dr1790*. After digestion with *Nde*I and *Bam*HI, the target gene *dr1790* was ligated into *Nde*I- and *Bam*HI-pre-digested pRADK, and the resulting construct was designated as pRAD-*dr1790*. The complementation plasmids were confirmed by PCR and DNA sequence analyses; thus, transformation into R1Δ*dr1790* generated the functional complementation strain mutant Dr1790com.

### Measurement of growth rate

The growth rate was measured as described previously (Mattimore *et al.*, 1995). Briefly, 500 μL overnight culture of each strain was transferred to 50 mL TGY medium. The culture was grown at 30 °C with agitation (200 rpm). Then, the culture dilutions were spread onto TGY agar plates after 2 (t_1_) and 4 h (t_2_). The plates were incubated at 30 °C for 5 days, and subsequently, the number of colony-forming units (CFU) was determined. The double time (*g*) was calculated according to the following formula: *g* = ln2 / ((log_10_N_2_ - log_10_N_1_) 2.303/Δt), where N_1_ is CFU per milliliter at t_1_, and N_2_ is CFU per milliliter at t_2_.

### Cell survival under oxidative stress and ionizing radiation

The hydrogen peroxide sensitivity of *D. radiodurans* cells was assayed as described previously (Wang *et al.*, 2008), with some modifications. The cells were harvested during the early stationary phase (OD_600nm_ = 1.0), washed twice and re-suspended with phosphate buffer (20 mM, pH 7.4). An aliquot was removed as a control, and the remaining suspension was treated with hydrogen peroxide at a final concentration of 20 mM. The mixture was incubated at 30 °C in an orbital shaker. Catalase (Sigma-Aldrich) was added in excess (15 U) to terminate the H_2_O_2_ treatment. Then, the cells were diluted and spread onto solid TGY media to determine the number of CFUs. The survival fractions were defined as a percentage of the CFU obtained in the treated sample compared with the control. The data are presented as the means ± SD of three independent experiments.

The cell survival fractions under ionizing radiation were determined using a previously described method (Wang *et al.*, 2008).

### Measurement of protein carbonylation levels

Protein carbonylation, which is an indicator of intracellular protein oxidation, was measured using the DNPH (2,4-dinitrophenyl hydrazine) method (Tian *et al.*, 2009).

### Membrane localization of the DR1790 protein

The plasmid pRADG-*dr1790* was constructed as described previously (Gao *et al.*, 2008). pRADG-*dr1790* was transformed into the R1Δ*dr1790* mutant strain. The transformant was obtained by chloramphenicol-resistance selection. The transformant was grown to the exponential phase (OD_600nm_ is approximately 0.8), spread on a glass slide and examined using a laser confocal microscope (Zeiss LSM510, Germany).

### Membrane integrity assessment

Differences in membrane permeability between the varying strains were assessed using a LIVE/DEAD BacLight Bacterial Viability Kit (Invitrogen, Carlsbad, CA, USA). This system employs two nucleic acid stains: green-fluorescent SYTO9 stain and red-fluorescent propidium iodide (PI) stain. Live cells with intact membranes fluoresced green, while dead cells or cells with compromised membranes fluoresced red. Bacterial cells were grown to mid-exponential phase, and a 1-mL aliquot of the culture was normalized to an OD_600nm_ equal to 0.6, washed twice with PBS, and resuspended in 1 mL PBS. The bacterial suspensions were stained with the nucleic acid dyes according to the manufacturer's protocol; then, 10 μL stained bacteria was spotted onto glass coverslips and visualized using a Leica DM4000B wide-field epifluorescence microscope (Leica Microsystems, Wetzlar, Germany). In total, 10 different fields were viewed for each strain, and the numbers of green, red or mixed cells were counted for each field.

### Transcriptome analysis

The procedures used for microarray hybridization and data analyses were performed as described previously (Chen *et al.*, 2008). Briefly, total RNA was prepared from three replicates of wild type and R1Δ*dr1790* mutant cells. Approximately 16 μg total RNA was annealed with 10 μg random hexamer primers in a total volume of 20 μL at 70 °C for 10 min, followed by incubation on ice for 2 min. cDNA synthesis was performed at 42 °C overnight in a 31-μL reaction mixture using SuperScript III Reverse Transcriptase (Invitrogen) with 0.5 mM dNTP mix containing amino allyl-dUTP (GE, Piscataway, NJ, USA). The reaction was terminated by adding 20 μL 0.5 M EDTA and 20 μL 1 M NaOH, followed by heating at 65 °C for 20 min. The reaction mixture was neutralized with 50 μL 1 M HEPES buffer (pH 7.0), and unincorporated amino allyl-dUTPs were removed by ultra-filtration using YM 30 columns (Millipore). The cDNA was coupled to 1 pmol Cy3 or Cy5 dye (GE) in 0.1 M sodium carbonate buffer for 2 h at room temperature, and free Cy3 or Cy5 was removed. The labeled pools of wild type and R1Δ*dr1790* mutant cDNA were mixed and simultaneously hybridized with the DNA chips in a solution containing 3X saline sodium citrate (SSC), 0.3% SDS, and 24 μg unlabeled herring sperm DNA (Gibco BRL, Gaithersburg, MD, USA). Normalization and statistical analysis were performed in the R computing environment (version 2.2.0, R with Aqua for Windows) using the linear modeling features of the Limma microarray data package (Wettenhall *et al.*, 2004). Before channel normalization, microarray outputs were filtered using Limma to remove spots with poor signal quality by excluding data points with a mean intensity less than two standard deviations above the background in both channels. Then, global LOESS normalization was used to normalize all data, and three replicate spots per gene in each array were used to maximize the robustness of the differential expression measurement of each gene via the "lmFit" function. The transcriptome analysis data were deposited in the Gene Expression Omnibus database under accession no. GSE22628.

### Real-time quantitative PCR

The genes of interest were identified using real-time quantitative PCR to validate the results of the microarray data. *dr0089*, which is a gene whose expression is unaffected by H_2_O_2_ and ionizing radiation, was used for normalization. Briefly, first-strand cDNA synthesis was performed in 20-μL reactions containing 1 μg each DNase I-treated and purified total RNA sample and 3 μg random hexamers. The real-time PCR amplification was performed using a Toyobo SYBR Green I Real Time PCR kit (Japan) according to the manufacturer's instructions under the following conditions: 94 °C for 2 min, followed by 40 cycles at 94 °C for 10 s, annealing at 56–62 °C for 15 s and 72 °C for 30 s. All assays were performed using a Stratagene Mx3005P qPCR system (Stratagene, Cedar Creek, TX, USA).

### Proteomics analysis

Proteomic analysis of the mutant compared with the wild type strain was performed using 2-DE and data analyses, in-gel digestion, MALDI-TOF MS analysis and a PMF spectra-based database search (Lu *et al.*, 2009).

## Results

### Growth of the deletion mutant

**Figure 1:**
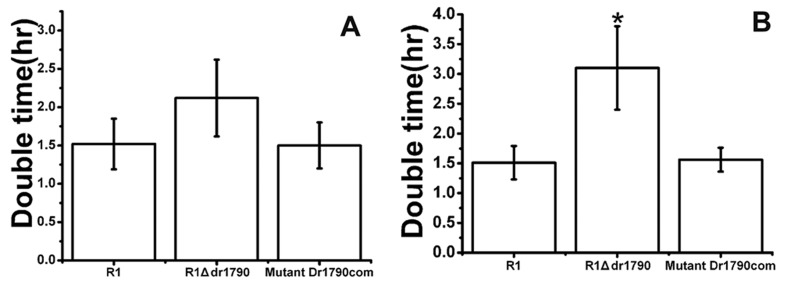
Growth of wild type *D. radiodurans* R1 compared with the R1Δ*dr1790* mutant strain under normal conditions in the lag (A) and log (B) phases. The error bars represent the standard deviations from three experiments.

We assayed the doubling time of cells in the lag and log phases. The R1Δ*dr1790* mutant doubling time (2.1 ± 0.4 h) was not significantly slower than the doubling time (1.5 ± 0.4 h) of the wild type R1 strain under aerobic conditions during the lag phase (*p* > 0.05) (Figure 1A). However, the R1Δ*dr1790* mutant doubling time (3.1 ± 0.5 h) was slightly slower than the doubling time (1.6 ± 0.2 h) of the wild type R1 strain under aerobic conditions during the log phase (*p* < 0.05) (Figure 1B).

### The deletion mutant was sensitive to oxidative stress and radiation

**Figure 2:**
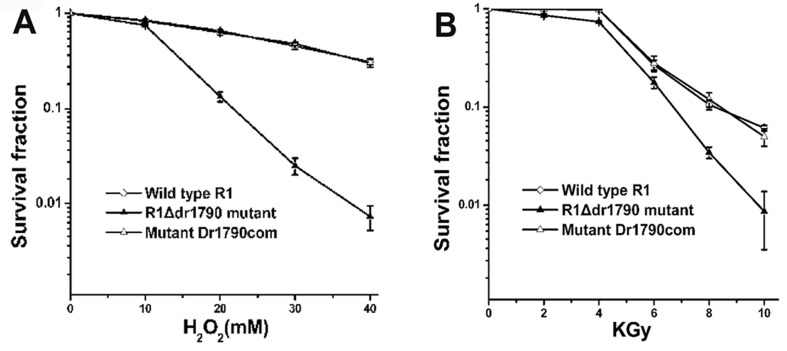
Survival curves for *D. radiodurans* following exposure to H_2_O_2_ (A) and gamma radiation (B). Each data point represents the mean of three replicates (bars indicate the standard deviations).

The yellow-related protein homolog DR1790 from *D. radiodurans* functions has been implicated in cell resistance to oxidative stress and radiation. The R1Δ*dr1790* mutant was sensitive to hydrogen peroxide treatment and γ-ray radiation. Compared with the wild type strains, the survival of the R1Δ*dr1790* mutant cells decreased nearly 15-fold in response to 30 mM hydrogen peroxide (Figure 2A) and nearly 3-fold in response to a 8 kGy dose (Figure 2B).

To determine whether the loss of DNA damage tolerance in the R1Δ*dr1790* mutant reflected the absence of *dr1790* and not a polar effect of this mutation, the wild type allele of this gene was cloned into pRADgro, which is a *Deinococcus* expression vector, and the protein was expressed in R1Δ*dr1790* mutant cells. The radiation and oxidative resistance of the complemented mutant Dr1790com strain nearly recovered to the phenotype of the wild type strain (Figure 2), suggesting that the sensitivity of the R1Δ*dr1790* mutant reflected the absence of the *dr1790* gene.

### Comparison of intracellular protein oxidation levels between the wild type R1 strain and the R1Δ*dr1790* mutant strain

**Figure 3:**
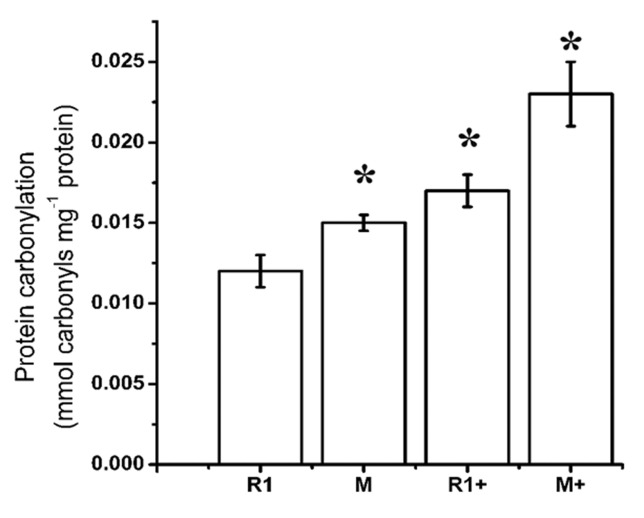
Comparison of the intracellular protein carbonylation levels between wild type R1 and R1Δ*dr1790* mutant strains following H_2_O_2_ treatment. R1 and M represent wild type R1 and R1Δ*dr1790* mutant strains under normal conditions, respectively. R1+ and M+ represent wild type R1 and R1Δ*dr1790* mutant strains following H_2_O_2_ treatment, respectively. Each data point represents the mean of three replicates (bars indicate the standard deviations). The results were assessed using Student's *t*-test, and statistical significance was considered at p < 0.05.

The level of protein oxidation in the R1Δ*dr1790* mutant was analyzed and compared with that of the wild type R1 strain (Figure 3). The total protein carbonyl contents measured in the wild type and mutant strains were 0.012 and 0.015 mmol/mg, respectively, indicating that the mutant strain exhibited relatively higher levels of protein oxidation compared with the wild type strain in the absence of oxygen stress. Following H_2_O_2_ treatment, intracellular protein carbonylation significantly increased in both the wild type and R1Δ*dr1790* mutant strains. The carbonyl content in the R1Δ*dr1790* mutant post-H_2_O_2_ treatment was 0.023 mmol/mg protein, which was significantly higher than the content in the wild type cells (0.017 mmol/mg protein, *p* < 0.05), suggesting that the intracellular proteins in the mutant cells lacking DR1790 were more sensitive to oxidative damage than those in the wild type cells.

### Membrane localization of the DR1790 protein and membrane integrity of the R1Δ*dr1790* mutant strain

**Figure 4:**
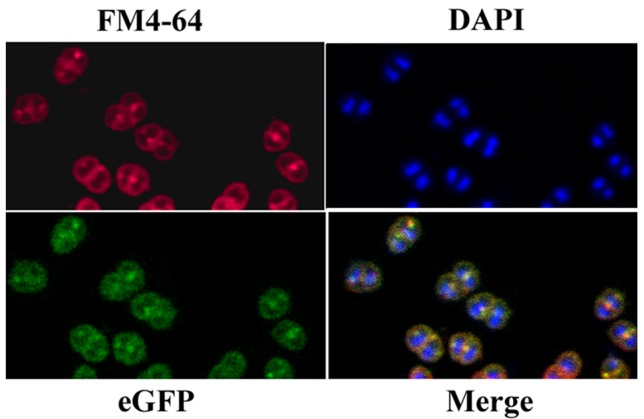
Analysis of DR1790 protein localization by fluorescence labeling. Images show FM4-64-stained membranes (red), DAPI-stained DNA (blue), eGFP-labeled proteins (green), and the merged image shows eGFP labeling and FM4-64 and DAPI staining (630).

Fusion gene expression of the green fluorescence protein (eGFP) gene and *dr1790* was performed and analyzed by fluorescence microscopy to confirm the localization of the DR1790 protein (Gao *et al.*, 2008). Figure 4 shows that eGFP-labeled protein (green fluorescence) was localized to the cell membrane; the yellow fluorescence displayed in the merged picture indicates the co-localization of eGFP-labeled proteins and FM4-64 (red fluorescence)-labeled membranes, confirming that DR1790 is a membrane protein.

**Figure 5:**
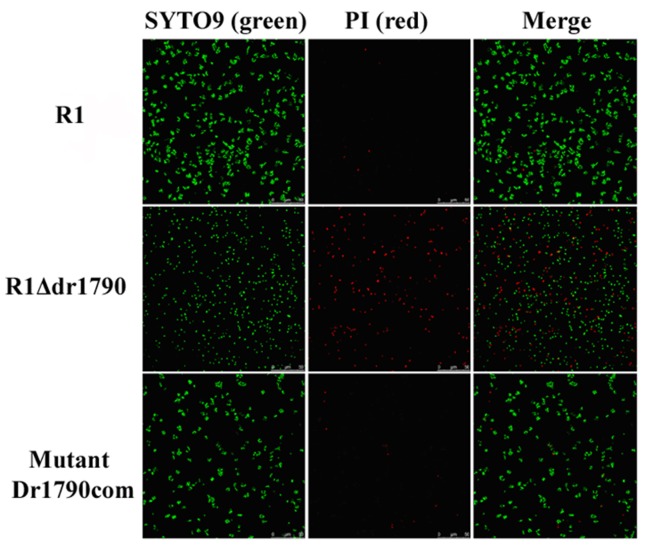
Stained images of wild type R1, R1Δ*dr1790* and complemented Mutant Dr1790com strains using a LIVE/DEAD kit (100). Live cells with intact membranes only incorporated SYTO9 (green), whereas dead cells or cells with compromised membranes incorporated PI (red).

The membrane integrity of the mutant strain was analyzed based on permeability assays using membrane-permeant and membrane-impermeant fluorescence-labeled nucleic acids. The R1Δ*dr1790* mutant incorporated both the membrane-impermeant dye propidium iodide (PI) and the membrane-permeant dye SYTO9 (Figure 5). Of 1384 mutant bacterial cells counted in 10 different fields, 20% of the cells incorporated PI (red). In contrast, wild type R1 and complemented mutant Dr1790com strains incorporated SYTO9 (green); however, only 1% of the cells were PI-positive among the 1464 wild type and 1538 complemented mutant bacteria that were counted in 10 independent fields (Figure 5). Thus, the R1Δ*dr1790* mutant showed a high proportion of damaged membranes (20% red cells observed in the mutant field) compared with wild type and complemented mutant strains. This result suggests that the DR1790 protein contributes to membrane permeability.

### Transcriptional and translational profiles of the DR1790 mutant *vs.* the wild type strain

**Figure 6:**
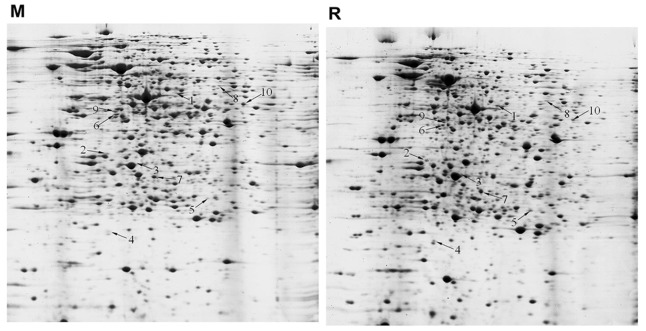
2-D gel images visualized by Coomassie Blue staining. The cells were analyzed by 2-DE and visualized by Coomassie Blue staining as described in the Materials and methods section. (M) R1Δ*dr1790* mutant, (R) R1.

2-DE and MS analyses were applied to compare the differential protein expression profiles of the R1Δ*dr1790* mutant and the wild type R1 strains (Figure 6). Ten protein spots showing two-fold changes in intensity in the R1Δ*dr1790* mutant compared with the wild type R1 were observed, including growth-related metabolism enzymes (IDH, MDH and FBP2), the predicted transmembrane protein transporter DR1909, and the chaperone protein DnaJ (Table 2). The limited information acquired by 2-DE analysis prompted the use of DNA microarray analysis to investigate this issue further.

**Table 2:** Mass spectrometry identification of the protein spots that were separated by 2-DE analysis.

Protein spot	Locus	Length (aa)	Functional category	Expression ratio, mutant 1790/WT (fold)
1	DR1540	430	Isocitrate dehydrogenase (IDH)	0.05
2	DR2013	268	Fructose 1,6-bisphosphatase II (FBP2)	0.09
3	DR0325	330	Malate dehydrogenase (MDH)	0.03
4	DR1512	264	Elongation factor Ts	0.09
5	DR0350	571	Serine/threonine protein kinase	0.29
6	DRA0337	386	Glutaryl-CoA dehydrogenase	2.49
7	DR1172	298	Cell envelope integrity inner membrane protein	0.18
8	DR1909	212	Predicted transmembrane protein transporter	0.09
9	DR1148	175	Putative TrkA-C domain protein Tyrosine kinase	2.56
10	DR0126	312	Chaperone protein DnaJ	3.39

The transcriptome of the R1Δdr1790 mutant was analyzed and compared with that of the wild type strain under normal growth conditions using oligonucleotide microarray to examine the expression of the entire gene repertoire of *D. radiodurans* in response to *dr1790* knockout. In the present study, a two-fold difference in the relative transcription level was selected as the threshold for microarray data analysis as described previously by Chen *et al.* (2008). We observed that 1.5% of the genes represented on the microarray (*n* = 46) were differentially transcribed in the R1Δ*dr1790* mutant compared with the WT. Among these genes, 27 were up-regulated (Table 3), and 19 were down-regulated (Table 3). These genes were involved in DNA/RNA repair, energy metabolism, various transporters, proteases and chaperones, stress responses, and translation and transcription functions.

**Table 3:** Summary of the gene expression results from microarray data. The 27 most highly repressed genes in the R1Δ*dr1790* mutant. The 19 most highly induced genes in the R1Δ*dr1790* mutant.

Locus	Annotation	Fold decrease	p value
DR1900	Predicted secreted protein	−23.35015	6.9E-06
DRB0006	Hypothetical protein	−6.61064	0.004
DR1702	NH2 acetyltransferase	−6.322434	0.005
DRB0045	Hypothetical protein	−5.757131	0.005
DR1085	SAM-dependent methyltransferase	−5.548009	0.005
DR0763	Acetyltransferase	−5.013164	0.006
DR1913	DNA gyrase, subunit A (gyrA)	−4.815689	0.011
DR2312	Carbohydrate kinase, PfkB family	−4.445003	0.007
DR1901	Predicted secreted protein	−3.834828	0.019
DR2625	Lipid A disaccharide synthase-related enzyme	−3.64618	0.009
DR2307	Multidrug-efflux transporter, putative	−3.451829	0.010
DR1912	Protein-tyrosine phosphatase, putative	−3.427269	0.011
DR1157	Hydroxypyruvate reductase, putative	−3.409991	0.010
DR2333	NADH oxidase-related protein	−3.152435	0.011
DR1591	Hypothetical protein	−3.149012	0.011
DR1481	Chlorite dismutase family enzyme	−2.949308	4E-03
DR2285	A-G-specific adenine glycosylase (mutY)	−2.799988	0.013
DRC0037	Nodulation protein-related protein	−2.666197	0.015
DRA0300	Predicted secreted protein	−2.620658	4E-03
DR2544	Predicted secreted protein	−2.604338	0.015
DRA0302	Hypothetical protein	−2.590681	2E-03
DR1916	DNA helicase RecG (recG)	−2.543044	0.011
DR1359	ABC-type metal ion transport system	−2.518423	0.060
DR2259	Transcriptional regulator	−2.45463	0.017
DRA0061	Permease MDR-type	−2.111218	0.023
DR0610	P-loop ATPase of adenylate kinase family	−2.086652	0.058
DR2213	Conserved hypothetical protein	−2.079364	0.024
Locus	Annotation	Fold increase	*p* value
DR0888	Distant homolog of OsmY	2.02588	5E-03
DR2403	Predicted membrane protein	2.03289	0.025
DR1306	Predicted secreted protein	2.03588	0.007
DRA0234	Hypothetical protein	2.04611	0.004
DR1114	HSP20	2.06477	0.005
DR0201	Hypothetical protein	2.08698	0.033
DRA0143	3-Hydroxyacyl-CoA dehydrogenase	2.08777	0.024
DR2385	Phenylacetic acid degradation protein PaaB	2.16397	0.008
DRA0290	Cell division protein FtsH (ftsH-3)	2.21667	0.007
DR0607	GroEL protein (groEL)	2.28587	0.001
DR1046	ATP-dependent Clp protease, ATP-binding subunit ClpB (clpB)	2.34619	0.012
DR0194	Predicted Zn-dependent protease	2.39411	0.003
DR0128	GrpE protein (grpE), HSP20 cofactor	2.46405	0.026
DR0129	DnaK protein (dnaK)	2.47161	8E-03
DRA0028	Hypothetical protein	2.47212	0.043
DRA0027	Putative L-lysine 2,3-aminomutase, Lysine degradation	2.54777	0.016
DR0126	DnaJ protein (dnaJ-1)	2.59145	0.003
DR1974	ATP-dependent protease LA (Lon1)	2.72752	0.003
DR0606	Chaperonin (groES)	2.76172	7E-03

Among the up-regulated genes in the R1Δ*dr1790* mutant, three genes were categorized as proteinase genes, six genes were related to protein quality control, and some genes encoded unknown/hypothetical proteins. Similarly, the down-regulated genes in the R1Δ*dr1790* mutant included four genes that were related to secreted proteins. The effectiveness of the microarray data was further confirmed by real-time quantitative RT-PCR (Table 4). Notably, many molecular chaperones and proteinases were positively regulated in the R1Δ*dr1790* mutant, and transporters and kinases were negatively regulated in the R1Δ*dr1790* mutant. These data demonstrate that the deletion of the *dr1790* gene significantly increased the amount of misfolded proteins in the cell*.* Some secreted proteins and transmembrane protein transporters were repressed, indicating that the DR1790 protein could be associated with secretory factors in the membrane.

**Table 4:** Real-time PCR relative quantification of the expression of repressed and induced genes in the R1Δ*dr1790* mutant compared with the *D. radiodurans* wild type strain.

Function	Gene name	Locus	Annotation	qRT-PCR Fold change
Heat, General	DnaJ-1	DR0126	HSP70 cofactor	2.13
	GrpE	DR0128	HSP20 chaperonin	2.45
	DnaK	DR0129	HSP70 chaperonin	2.53
	GroES	DR0606	Hsp10 chaperonin	2.18
	GroEL	DR0607	Hsp60 chaperonin	2.27
	Hsp20	DR1114	Molecular chaperone	4.57
General	HtpX	DR0194	Zn-dependent protease, Bacillus yugP ortholog	2.38
	ClpB	DR1046	ClpB, AAA superfamily ATPase	31.55
	Lon	DR1974	ATP-dependent Lon protease, bacterial type	4.68
Osmotic	OsmY	DR0888	Distant homolog of OsmY	4.86
Others		DR0350	Serine/threonine protein kinase	−2.14
		DR1172	Cell envelope integrity inner membrane protein	−2.32
		DR1909	Predicted transmembrane protein transporter	−1.14
		DR1148	Putative TrkA-C domain protein Tyrosine kinase	−2.83

## Discussion

The extreme resilience of *D. radiodurans* to oxidative and radiation stresses is imparted synergistically by the efficient protection of proteins against oxidative stress and efficient DNA repair mechanisms, enhanced by functional redundancies in both systems (Slade and Radman, 2011). Maleszka *et al.* (2000) identified an orphan protein (DR1790) in *D. radiodurans* belonging to the yellow-related protein family, which was originally identified in *Drosophila*. A mutation in the yellow-related protein in *Drosophila* affects the pigmentation of larvae and exerts some effects on insect behavior (Drapeau *et al.*, 2006). In the present study, the predicted yellow-related protein DR1790, which belongs to the ancient Yellow/MRJ protein family, was confirmed to be a membrane-binding protein. A null-mutant strain (R1Δ*dr1790*) exhibited reduced survival after gamma irradiation and H_2_O_2_ treatment, demonstrating that DR1790 is involved in the radioresistance and antioxidant mechanisms of *D. radiodurans*. Protein, rather than DNA, was suggested to be the principal target of radiation and free radicals, and the degree of cell resistance was determined based on the level of oxidative protein damage (Daly *et al.*, 2007). We observed that the total protein carbonyl contents increased in the R1Δ*dr1790* mutant under normal conditions and H_2_O_2_ treatment, demonstrating that the absence of DR1790 increased oxidative damage in cells.

Cellular membranes, which are composed of lipids, proteins, and carbohydrates, are damaged by radiation. The melting of membranes under stress results in permeability barrier loss and leakage, as well as the inability to maintain a proton gradient for respiration. The *D. radiodurans* cell envelope consists of at least five layers (Lancy *et al.*, 1978). *D. radiodurans* irradiated with 4 kGy loses up to 30% wet weight resulting from the loss of polysaccharides into the growth medium, which suggests permeability alterations in the cell envelope (Mitchel, 1976). For retaining membrane integrity, *D. radiodurans* cells were much more resistant to high temperatures when exposed in the dried state as opposed to cells in suspension (Bauermeister *et al.*, 2012). The R1Δ*dr1790* mutant showed a high proportion of damaged membranes (20% red cells observed in the mutant field) compared with wild type and complemented mutant strains. This result suggests that the DR1790 protein contributes to membrane permeability. Consequently, the mutant strain was more sensitive to both ionizing radiation and oxidative stress. However, how DR1790 contributes to bacterial membrane integrity remains unclear. DR1790 may be required for the stability of membrane protein complexes to restore the osmotic imbalance rapidly, and the absence of DR1790 may result in less stability or improperly gated channels or pores. Thus, the isolation of the protein partners of DR1790 may help to clarify the role of this protein in membrane homeostasis. Alterations in membrane integrity may also contribute to the increased sensitivity of R1Δ*dr1790* mutants to oxidative and radiation stresses.

In the present study, some genes that are involved in protein quality control, such as *dr1114* (HSP20), *dr0129* (dnaK), *dr0126* (dnaJ), *dr0607* (groEL), *dr1046* (ATP-binding subunit ClpB), and *dr1974* (ATP-dependent protease LA, Lon1), were strongly induced in R1Δ*dr1790* mutants. The induction of these chaperones and proteases suggested that many damaged proteins aggregated in the R1Δ*dr1790* mutant. Chaperones assist in non-covalent folding or unfolding and in the assembly or disassembly of protein structures in the cell, but do not occur in these structures during the performance of normal biological functions after having completed folding and/or assembly. DnaK/DnaJ/GrpE and GroEL/ES are the two primary chaperone foldase systems in prokaryotic cells (Hoffmann *et al.*, 2004). ATP-dependent proteases function in protein processing and play an essential role in diverse stress responses (Gottesman, 2003). In *D. radiodurans*, the majority of cellular proteolysis is performed by ATP-dependent proteases that belong to the Lon (Lon1 and Lon2) and Clp families (ClpA, ClpB, ClpC, ClpX and ClpP). The ClpPX protease is required for radioresistance and regulates cell division after γ-irradiation in *D. Radiodurans* (Servant *et al.*, 2007). ClpB from *Myxococcus xanthus* functions as a chaperone protein and plays an important role in cellular heat and osmotic stress tolerance mechanisms during both vegetative growth and development (Pan *et al.*, 2012). ClpB and the DnaK system act synergistically to remodel proteins and to dissolve aggregates (Doyle *et al.*, 2007). HSPs function as molecular chaperones that prevent protein denaturation and aggregation (Feder and Hofmann, 1999; Matuszewska *et al.*, 2008). Additionally, some genes that are involved in protein secretion and transport are strongly suppressed in R1Δ*dr1790* mutants, such as secreted proteins (DR1900, DR1901, DRA0300, and DR2544) and transmembrane transporter proteins (DR1909), indicating that the DR1790 protein could be related to secretory factors in the membrane. *D. radiodurans* contains many secreted proteases and transporters that provide exogenous amino acids as protein building blocks and peptides as components of manganese complexes (Slade and Radman, 2011). After irradiation in *D. radiodurans*, 10 secreted subtilisin-like proteases, and 4 peptide and amino acid ABC transporters were highly induced (Makarova *et al.*, 2000; Ghosal *et al.*, 2005). Thus, the low growth rate and sensitivity to hydrogen peroxide and radiation in the R1Δ*dr1790* mutant were closely associated with the induction of these chaperones and proteases and with the suppression of secreted and transported proteins. Additionally, the expression of some genes involved in antioxidant systems, electron transport, and energy metabolism were also significantly altered by the disruption of DR1790.

In conclusion, we presented the first experimental evidence that a protein from the ancient yellow protein family plays a role in the survival of prokaryote cells during a damage response. The DR1790 protein from the ancient yellow protein family plays a pleiotropic role in the survival of prokaryotic cells and contributes to the extraordinary resistance of *D. radiodurans* against oxidative and radiation stresses. Further studies are required to understand the mechanisms of the action that are mediated by DR1790 during this process and to identify critical protein interactions.
